# Loss of Upk1a and Upk1b expression is linked to stage progression in urothelial carcinoma of the bladder

**DOI:** 10.1007/s11255-023-03800-0

**Published:** 2023-10-01

**Authors:** Krystian Kaczmarek, Henning Plage, Kira Furlano, Sebastian Hofbauer, Sarah Weinberger, Bernhard Ralla, Antonia Franz, Annika Fendler, Michela de Martino, Florian Roßner, Simon Schallenberg, Sefer Elezkurtaj, Martina Kluth, Maximilian Lennartz, Niclas C. Blessin, Andreas H. Marx, Henrik Samtleben, Margit Fisch, Michael Rink, Marcin Slojewski, Thorsten Ecke, Steffen Hallmann, Stefan Koch, Nico Adamini, Sarah Minner, Ronald Simon, Guido Sauter, Joachim Weischenfeldt, Tobias Klatte, Thorsten Schlomm, David Horst, Henrik Zecha

**Affiliations:** 1 Department of Urology and Urological Oncology, Pomeranian Medical University, Szczecin, Poland; 2 Department of Urology, Charité – Universitätsmedizin Berlin, Corporate Member of Freie Universität Berlin, Humboldt-Universität zu Berlin and Berlin Institute of Health, Berlin, Germany; 3 Institute of Pathology, Charité – Universitätsmedizin Berlin, Corporate Member of Freie Universität Berlin, Humboldt-Universität zu Berlin and Berlin Institute of Health, Berlin, Germany; 4 Institute of Pathology, University Medical Center Hamburg-Eppendorf, Martinistr. 52, 20246 Hamburg, Germany; 5 Department of Pathology, Academic Hospital Fuerth, Fuerth, Germany; 6 Department of Urology, University Medical Center Hamburg-Eppendorf, Hamburg, Germany; 7 Department of Urology, Marienhospital Hamburg, Hamburg, Germany; 8 Department of Urology, Helios Hospital Bad Saarow, Bad Saarow, Germany; 9 Department of Pathology, Helios Hospital Bad Saarow, Bad Saarow, Germany; 10 Department of Urology, Albertinen Hospital, Hamburg, Germany; 11 Biotech Research & Innovation Center (BRIC), University of Copenhagen, Copenhagen, Denmark; 12 Finsen Laboratory, Rigshospitalet, Copenhagen, Denmark

**Keywords:** Upk1a, Upk1b, Immunohistochemistry, Tissue microarray, Urothelial carcinoma, Diagnostic marker

## Abstract

**Background:**

Uroplakin-1a (Upk1a) and uroplakin-1b (Upk1b) have recently been identified as diagnostic markers for the distinction of urothelial carcinomas from other solid tumor entities. Both proteins play an important role in the stabilization and strengthening of epithelial cells that line the bladder.

**Methods:**

To evaluate the prognostic role of uroplakin expression in urothelial carcinomas, more than 2700 urothelial neoplasms were analyzed in a tissue microarray format by immunohistochemistry. To further assess the diagnostic role of uroplakin immunohistochemistry, results were compared with preexisting GATA3 data.

**Result:**

The fraction of Upk1a/Upk1b positive cases decreased slightly from pTaG2 low-grade (88% positive for Upk1a/87% positive for Upk1b) and pTaG2 high-grade (92%/89%) to pTaG3 (83%/88%; *p* > 0.05) and was lower in muscle-invasive (pT2-4) carcinomas (42%/64%; *p* < 0.0001/*p* < 0.0001 for pTa vs. pT2-4). Within pT2-4 carcinomas, high expression of Upk1a and Upk1b was linked to nodal metastasis and lymphatic vessel infiltration (*p* < 0.05) but unrelated to patient outcome. There were significant associations between Upk1a, Upk1b and GATA3 immunostaining (*p* < 0.0001 each), but 11% of GATA3 negative cancers were Upk1a/b positive and 8% of Upk1a/b negative cancers were GATA3 positive. Absence of GATA3/Upk1a/b staining was significantly linked to poor patient survival in the subgroup of 126 pT4 carcinomas (*p* = 0.0004) but not in pT2 and pT3 cancers.

**Conclusions:**

In summary, the results of our study demonstrate that Upk1a and/or Upk1b immunohistochemistry can complement GATA3 for the distinction of urothelial carcinomas. Furthermore, a progressive loss of Upk1a/b expression during stage progression and a prognostic role of the combination GATA3/Upk1a/Upk1b in pT4 carcinomas is evident.

**Supplementary Information:**

The online version contains supplementary material available at 10.1007/s11255-023-03800-0.

## Introduction

Urinary bladder cancer is the tenth most common malignant tumor type worldwide [1]. About 80% of patients present with low-grade non-invasive (pTa) or minimally invasive (pT1) cancers, which are characterized by a good prognosis and can be removed by transurethral resection. However, more than 60% of these tumors recur and about 20% will further progress to life threatening muscle-invasive disease requiring surgical removal of the bladder [2]. In patients with muscle-invasive bladder cancer, the clinical outcome is highly variable, but almost 50% of patients develop early metastasis and eventually die from their disease [3]. A better understanding of the molecular features underlying disease progression will eventually enable a better prediction of the individual patient prognosis and thus optimize treatment decisions and allow an early aggressive treatment in curable patients at high risk.

Uroplakin 1A (Upk1a) and uroplakin 1B (Upk1b) are two out of five known uroplakin proteins (Upk) that cooperatively form apical asymmetrical unit membrane (AUM) plaques (summarized in [4]). AUM plaques play an important role in the stabilization and strengthening of epithelial cells that line the bladder and enable the inner bladder membrane to stretch and prevent urothelial cells from rupturing during bladder distension [5]. Upk1a heterodimerizes with Upk2 and Upk1b with Upk3 [6]. Upk heterodimers subsequently form heterotetramers which then combine as concentric hexameric rings that are packaged into vesicles and trafficked to the cell surface (summarized in [4]). AUM plaques and Upk proteins may also have a role in mediating membrane permeability and signal transduction events that are involved in the regulation of cell development, activation, growth, and motility (summarized in [4, 7]). Upk1a has been identified as a receptor for uropathogenic Escherichia coli [8]. In recent studies on more than 6500 tumors from more than 100 different cancer types, Upk1a and Upk1b have been identified as potential markers for the distinction of urothelial carcinomas from other tumor entities [9, 10]. Studies evaluating the prognostic role of these proteins are lacking, however.

We thus studied the relationship between Upk1a and Upk1b immunostaining and clinicopathological parameters of disease progression as well as patient outcome in more than 2700 urothelial carcinomas in a tissue microarray (TMA) format. In addition, a comparison with GATA3 expression was conducted to further evaluate the diagnostic potential of Upk1a and Upk1b for identifying cancers of urothelial origin.

## Materials and methods

### Tissue microarrays (TMA)

The TMA method allows the analysis of a large number of molecular-genetic alterations on one TMA set. The TMAs used in this study were first employed in a study on the prognostic role of GATA3 expression in bladder cancer [11]. The TMA set contained one sample each from 2710 urothelial bladder tumors archived at the Institute of Pathology, University Hospital Hamburg, Germany, Institute of Pathology, Charité Berlin, Germany, Department of Pathology, Academic Hospital Fuerth, Germany, or Department of Pathology, Helios Hospital Bad Saarow, Germany, and/or treated at Department of Urology, University Hospital Hamburg, Germany, Department of Urology, Charité Berlin, Germany, Department of Urology, Helios Hospital Bad Saarow, Germany, Department of Urology, Albertinen Hospital, Hamburg, Germany, and Department of Urology and Urological Oncology, Pomeranian Medical University, Szczecin, Poland. Patients at each center were treated according to the guidelines at the time. In brief, patients with pTa/pT1 disease underwent a transurethral bladder tumor resection with or without postoperative or adjuvant instillation therapy. Patients with pT2-pT4 disease were treated by radical cystectomy. Available histopathological data including tumor stage (pT), grade, status of venous (V) and lymphatic (L) invasion, and lymph node status (pN) are shown in Supplementary Table 1. Clinical follow-up data (overall survival; OS: time between cystectomy and death) were available from 636 patients with pT2-4 carcinomas treated by cystectomy (median: 15 months; range: 1–176 months). Data on GATA3 immunostaining were available from a previous study for 2443 cancers [11]. All tissues were fixed in 4% buffered formalin and then embedded in paraffin. The TMA manufacturing process has previously been described in detail [12, 13]. In brief, one tissue spot (diameter: 0.6 mm) was transmitted from a cancer containing donor block in an empty recipient paraffin block. The use of archived remnants of diagnostic tissues for TMA manufacturing, their analysis for research purposes, and patient data were according to local laws (HmbKHG, §12) and analysis had been approved by the local ethics committee (Ethics commission Hamburg, WF-049/09). All work has been carried out in compliance with the Helsinki Declaration.

### Immunohistochemistry

For this study, we used identical methods for immunohistochemical evaluation of Upk1a and Upk1b as previously described [9, 10]. Freshly cut TMA sections were immunostained on one day and in one experiment. Slides were deparaffinized with xylol, rehydrated through a graded alcohol series, and exposed to heat-induced antigen retrieval for 5 min in an autoclave at 121 °C in a pH 7.8 DakoTarget Retrieval Solution^™^ (Agilent, CA, USA). Endogenous peroxidase activity was blocked with Dako Peroxidase Blocking Solution^™^ (Agilent, CA, USA; #52,023) for 10 min. A primary antibody specific for Upk1a (mouse monoclonal, MSVA-735 M, MS Validated Antibodies, Hamburg, Germany, 4386-735 M) or Upk1b (mouse monoclonal, MSVA-734 M, MS Validated Antibodies, Hamburg, Germany, 3797-734 M) were applied at 37 °C for 60 min at a dilution of 1:150. Bound antibody was then visualized using the EnVision Kit (Agilent, CA, USA; #K5007) according to the manufacturer’s directions. For the tumor TMA, the percentage of positive neoplastic cells was estimated for each tissue spot, and the staining intensity was recorded as 0, 1 + , 2 + , and 3 + . For statistical analyses, the tumor staining results were categorized into four groups. Tumors without any staining were considered negative. Tumors with 1 + staining intensity in ≤ 70% of cells or 2 + intensity in ≤ 30% of cells were considered weakly positive. Tumors with 1 + staining intensity in > 70% of cells, or 2 + intensity in 31%–70%, or 3 + intensity in ≤ 30% were considered moderately positive. Tumors with 2 + intensity in > 70% or 3 + intensity in > 30% of cells were considered strongly positive.

### Statistics

Statistical calculations were performed with JMP 16 software (SAS Institute Inc., NC, USA). Contingency tables were created, and Chi^2^-tests were performed to test for associations between Upk1a/Upk1b immunostainings as well as GATA3 and pathological parameters. Survival curves were calculated according to Kaplan–Meier. The Log-Rank test was applied to detect significant differences between groups.

## Results

### Technical issues

For Upk1a IHC, 2474 (91.6%) and for Upk1b 2515 (92.8%) of 2710 urothelial carcinomas were interpretable. Non-informative spots were caused by a lack of unequivocal tumor cells on the TMA spots or absence of entire tissue spots on the TMA.

### Upk1a in urothelial carcinomas

**Fig. 1:**
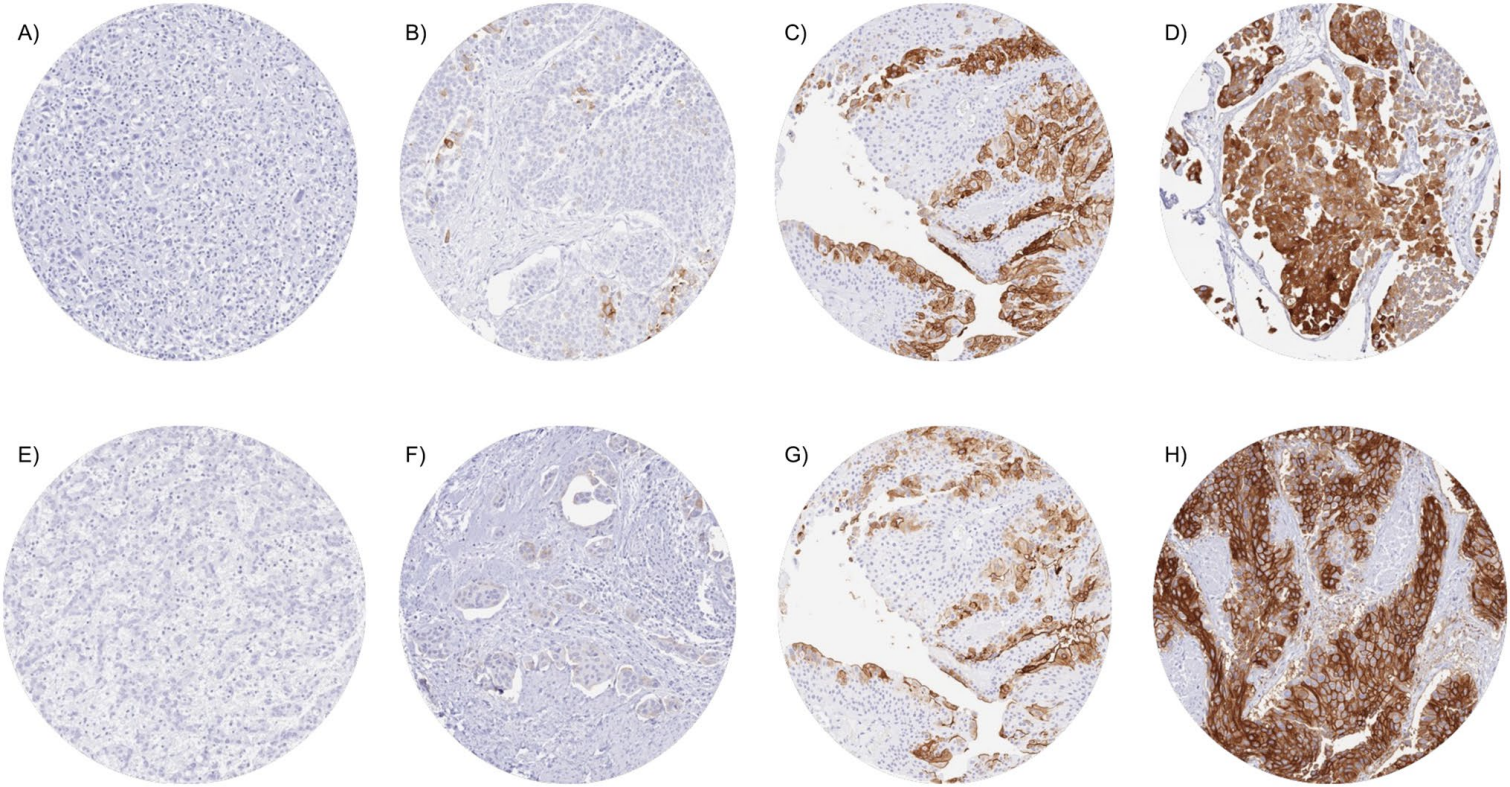
Upk1a and Upk1b immunostaining in urothelial carcinoma. The panels show a cytoplasmic and membranous positivity of variable intensity for Upk1a and Upk1b. Upk1a staining was absent (**A**), weak (**B**), moderate (**C**), and strong (**D**) in individual pT2-4 cases. Upk1b staining was absent (**E**), weak (**F**), moderate (**G**), and strong (**H**) in other examples from invasive urothelial carcinomas

**Fig. 2:**
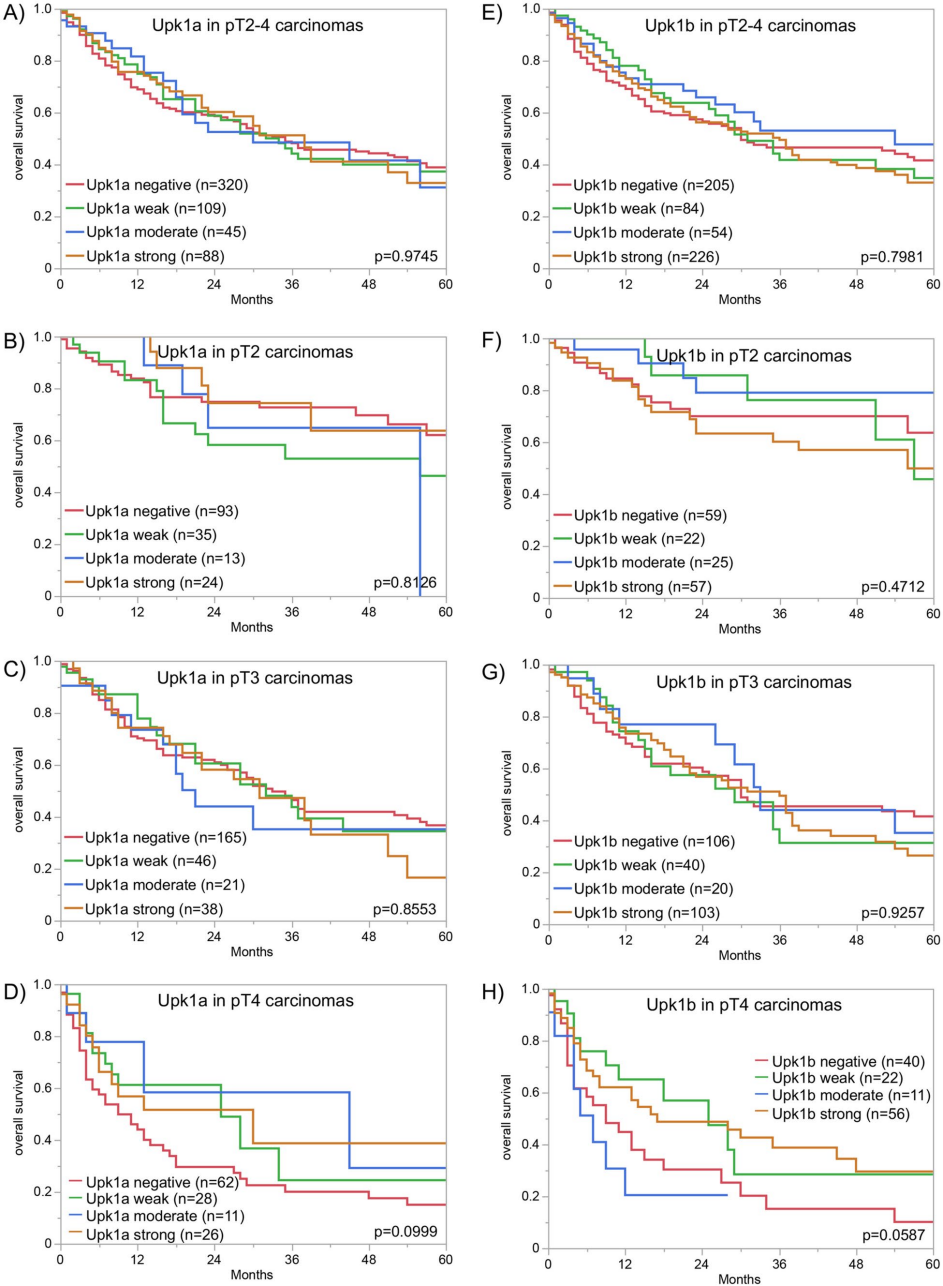
Uroplakin immunostaining and patient prognosis in muscle-invasive urothelial carcinomas. **A**–**D** Upk1a and overall survival, **E**–**H** Upk1b and overall survival

A cytoplasmic and membranous cancer cell staining was seen in 1400 (56.6%) of the 2474 interpretable cancers, including 569 (23.0%) with weak, 314 (12.7%) with moderate, and 517 (20.9%) with strong immunostaining. Representative images are shown in Fig. 1. Upk1a staining was highest in pTaG2 tumors (89.6%), insignificantly lower in pTaG3 tumors (83.2%, *p* = 0.1569), but markedly lower in muscle-invasive carcinomas (41.8%, *p* < 0.0001 for pTa vs. pT2-4; Table 1). Within muscle-invasive carcinomas, a statistically significant association between Upk1a expression and pT stage (*p* = 0.0295) was driven by a particularly low rate of Upk1a positivity in pT3 carcinomas (35.6%) while pT2 (43.6%) and pT4 (47.8%) cancers had higher positivity rates. Accordingly, Upk1a expression was unrelated to patient prognosis if the entire cohort was analyzed (Fig. 2A). A subset analysis revealed a tendency towards a favorable prognosis in pT4 carcinomas with high Upk1a levels (Fig. 2B; *p* = 0.0999) while such an association was not seen in pT2 and pT3 cancers (Fig. 2B, C). Upk1a positivity was also linked to nodal metastasis (*p* = 0.0092) and lymphangiosis carcinomatosa (L1; *p* = 0.0004).

**Table 1:** Upk1a and Upk1b immunostaining and cancer phenotype

	*n*	Upk1a immunostaining result				*p*-value	*n*	Upk1b immunostaining result				*p*-value
		Negative (%)	Weak (%)	Moderate (%)	Strong (%)			Negative (%)	Weak (%)	Moderate (%)	Strong (%)	
All cancers	2474	43.4	23.0	12.7	20.9		2515	28.6	15.0	10.4	46.0	
pTa G2 low	434	11.8	35.5	24.2	28.6	0.1596	447	12.3	9.8	12.8	65.1	0.1745
pTa G2 high	208	7.7	36.1	21.2	35.1		216	10.6	13.9	12.5	63.0	
pTa G3	143	16.8	31.5	21.7	30.1		146	11.6	18.5	14.4	55.5	
pT2	408	56.4	18.4	8.1	17.2	0.0295	419	37.0	14.1	10.5	38.4	0.7039
pT3	565	64.4	14.9	7.1	13.6		566	36.6	17.3	9.0	37.1	
pT4	278	52.2	19.1	8.6	20.1		283	35.3	15.2	8.1	41.3	
G2	110	55.5	16.4	9.1	19.1	0.8354	114	29.8	17.5	8.8	43.9	0.4421
G3	1545	58.4	17.4	8.0	16.2		1556	36.8	16.3	9.3	37.6	
pN0	645	61.6	16.1	7.8	14.6	0.0092	653	40.3	16.8	9.5	33.4	< 0.0001
pN +	421	51.3	19.5	9.0	20.2		419	28.9	15.0	9.1	47.0	
R0	525	59.6	17.0	7.4	16.0	0.8014	528	36.7	15.3	10.0	37.9	0.9726
R1	122	54.9	18.0	9.0	18.0		128	35.2	16.4	9.4	39.1	
L0	228	66.2	15.4	6.1	12.3	0.0004	237	41.4	19.4	11.0	28.3	0.0002
L1	253	47.8	20.2	8.7	23.3		255	29.8	14.9	7.5	47.8	
V0	393	61.6	16.8	6.4	15.3	0.0289	398	37.4	17.3	9.8	35.4	0.0843
V1	137	48.2	18.2	10.9	22.6		141	28.4	14.2	10.6	46.8	

*pT* Pathological tumor stage, *G* grade, *pN* pathological lymph node status, *R* resection margin status, *L* lymphatic invasion, *V* venous invasion

*Only in pT2-4 urothelial carcinoma

### Upk1b in urothelial carcinomas

**Fig. 3:**
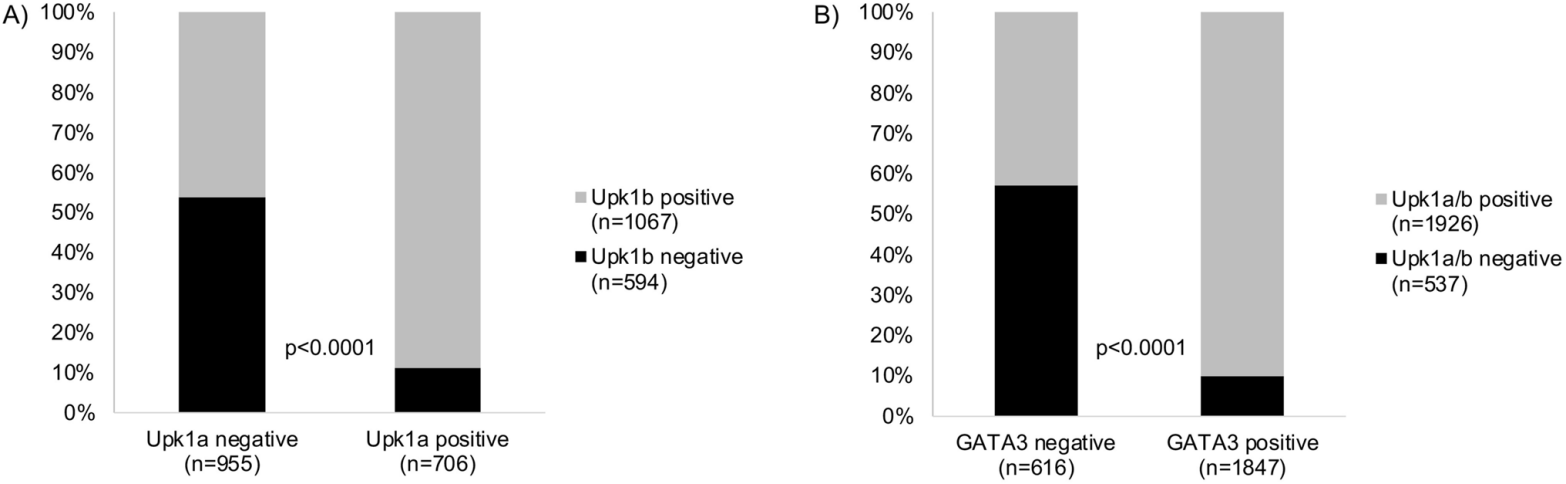
Comparison of **A** Upk1a and Upk1b immunostaining and **B** Upk1a/Upk1b and GATA3 immunostaining

Upk1b staining (cytoplasmic and membranous) was detectable in 1795 (71.4%) of 2515 interpretable cancers, including 377 (15.0%) with weak, 262 (10.4%) with moderate, and 1156 (46.0%) with strong staining. Representative images are shown in Fig. 1. Upk1b staining was highest in pTaG2 tumors (88.2%) and in pTaG3 tumors (88.4%) but markedly lower in muscle-invasive carcinomas (63.4%, *p* < 0.0001 for pTa vs. pT2-4; Table 1). Within the entire cohort of pT2-4 cancers, Upk1b staining was unrelated to pT status, histologic grade, and clinical disease course (Fig. 2E) but high Upk1b expression was significantly associated with L1-status (*p* = 0.0002) and nodal metastasis (*p* < 0.0001; Table 1). Upk1b expression was unrelated to patient prognosis both in the analysis of all pT2-4 carcinomas and in subgroups of pT2, pT3 and pT4 cancers (Fig. 2). There was a tendency, however, towards a favorable outcome in pT4 cancers with Upk1b positivity (Fig. 2H, p = 0.0587). Upk1b positivity was strongly linked to Upk1a staining. Within pT2-4 carcinomas, only 11.2% of 706 Upk1a positive cancers were Upk1b negative while 41.2% of 1,076 Upk1b positive cancers were Upk1a negative (*p* < 0.0001; Fig. 3A). The combined analysis of Upk1a and Upk1b revealed a link between high Upk1a/Upk1b expression and favorable patient prognosis in pT4 (*p* = 0.0365) but not in pT2 and pT3 carcinomas (Supplementary Fig. 1).

### Comparison with GATA3 immunostaining

**Fig. 4:**
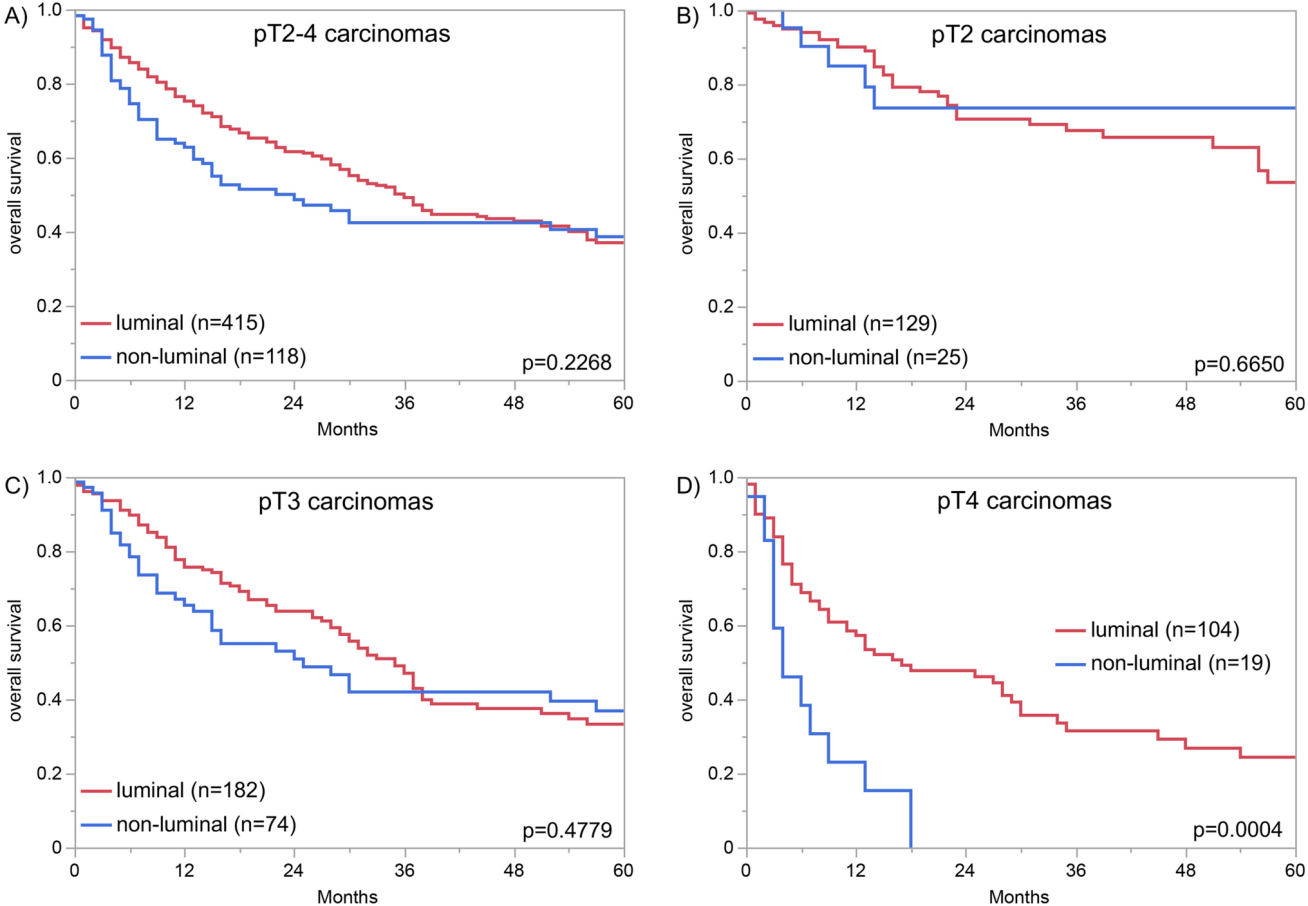
Combined Upk1a, Upk1b, and GATA3 immunostaining and patient prognosis. Luminal: immunostaining in at least one of Upk1a/Upk1b or GATA3; non-luminal: “triple negative” tumors—no immunostaining of Upk1a/Upk1b and GATA3

Given the strong association between Upk1a and Upk1b immunostaining, these two parameters were combined for a comparison with GATA3 expression. GATA3 and Upk1a/Upk1b immunostaining showed a strong statistical correlation in our 1596 pT2-4 carcinomas with data on all three markers (*p* < 0.0001). However, GATA3 positivity was found in 31.5% of 476 Upk1a/Upk1b negative while Upk1a/Upk1b positivity was observed in 42.7% of 569 GATA3 negative pT2-4 cancers (Fig. 3B). Example tumors with discrepant GATA3 and Upk staining are shown in Supplementary Fig. 2. After categorization of tumors with detectable immunostaining for at least one of Upk1a/Upk1b or GATA3 as luminal and of “triple negative” tumors as non-luminal, the luminal phenotype was associated with nodal metastasis (*p* = 0.0035), positive lymph vessel status (*p* = 0.0019), and blood vessel invasion (*p* = 0.0278) in pT2-4 carcinomas (Table 2). Associations with clinical outcome were not observed if the entire cohort was analyzed (Fig. 4A). There was, however, a significant link between luminal phenotype and favorable patient prognosis in pT4 (*p* = 0.0004, Fig. 4D) but not in pT2 and pT3 carcinomas (Fig. 4B and C).

**Table 2:** Combined GATA3 and Upk1a/Upk1b immunostaining and tumor phenotype

	GATA3/Upk1a/Upk1b immunostaining result			
	*n*	Luminal (%)	Non-luminal (%)	*p*-value
All cancers	2463	85.7	14.3	
pTa G2 low	432	99.8	0.2	0.2722
pTa G2 high	205	99.5	0.5	
pTa G3	131	98.5	1.5	
pT2	383	83.3	16.7	0.0005
pT3	533	73.4	26.6	
pT4	260	81.9	18.1	
G2	83	81.4	18.6	0.6739*
G3	1163	79.7	20.3	
pN0	605	74.9	25.1	0.0035*
pN +	397	82.6	17.4	
R0	491	78.2	21.8	0.2882*
R1	115	82.6	17.4	
L0	216	72.2	27.8	0.0019*
L1	240	84.2	15.8	
V0	370	76.5	23.5	0.0278*
V1	130	85.4	14.6	

luminal: GATA3, Upk1a or Upk1b positive; non-luminal: “triple negative”; *pT* pathological tumor stage, *G* grade, *pN* pathological lymph node status, *R* resection margin status, *L* lymphatic invasion, *V* venous invasion

*Only in pT2-4 urothelial carcinoma

## Discussion

The results of our study confirm a diagnostic utility of Upk1a and Upk1b IHC for the distinction of urothelial carcinomas from other tumor entities and also suggest a prognostic utility of Upk1a/Upk1b measurement as a part of a panel that distinguishes a “luminal” urothelial cancer phenotype.

Both Upk1a and Upk1b were recently identified as useful IHC markers for the distinction of urothelial carcinomas from its morphological differential diagnoses in studies investigating 6929 [9] and 14,061 [10] tumors from more than 110 different cancer entities. An IHC confirmation of urothelial tumor origin is particularly needed in solid cancer metastases of unknown origin and in solid carcinomas of the bladder base. In the latter situation, a morphological distinction of urothelial carcinoma from a poorly differentiated prostatic adenocarcinoma can often not be safely made in the absence of urothelial carcinoma in situ or another unequivocal precursor lesion of urothelial neoplasms (summarized in [14]).

GATA3 is the most commonly used IHC marker for the identification of urothelial carcinomas [15]. GATA3 expression occurs in 49%–100% of pT2-4 urothelial carcinomas but is also seen in 42%–100% of breast cancers, in salivary gland neoplasms and–less commonly—also in various other tumor types [11, 16–18]. The main limitation of GATA3 as a urothelial cancer marker comes from its lack of expression in almost a third of muscle invasive cancers. Our comparative analysis of Upk1a, Upk1b, and GATA3 immunostaining data revealed a high complementarity of these markers. Upk1a/Upk1b staining was observed in 32% of GATA3 negative pT2-4 cancers and GATA3 staining was seen in 43% of Upk1a/Upk1b negative pT2-4 urothelial carcinomas. It is also of note that Upk1a/Upk1b staining is only very rarely seen in breast cancer (0%–4%) and in salivary gland tumors (0%–10%) [9, 10]. We consider our Upk1a and Upk1b assays as highly suitable because both antibodies had earlier been validated according to the guidelines of the international working group for antibody validation (IWGAV) [19] by comparison with a second independent antibody and with RNA expression data obtained from three different publicly accessible databases in 76 different normal tissue categories [20–22].

Upk1a and Upk1b are always expressed in normal urothelium [9, 10]. The marked decrease of both prevalence and intensity of Upk1a and Upk1b immunostaining from non-invasive (pTa) to muscle-invasive carcinomas (pT2-4) reflects a striking loss of Upk expression during bladder cancer progression. Neoplastic transformation and tumor progression is often accompanied by a continuous loss of proteins that are physiologically expressed in non-neoplastic precursor cells [23–26]. While some alterations of the protein expression profile of cancer cells may have a “driving” role for cancer progression, it is believed that most of them do reflect non-functional “bystander” effects (summarized in [27]). A non-functional role of Upk1a/Upk1b loss is conceivable given the function of the protein for the strengthening of surface epithelial cells to allow epithelial distension (summarized in [4]). Most likely this function is not needed in cancer cells exhibiting solid tumor growth largely lacking exposure to mechanical stress.

A particular clinical interest in uroplakin expression of urothelial neoplasms is based on recent RNA expression studies suggesting molecularly defined urothelial carcinoma subgroups with distinct differences in prognosis (summarized in [28]) and perhaps also in response to specific treatments [29]. Together with GATA3, the expression of uroplakins has been described as a hallmark of the luminal subtypes which were characterized by a particularly good patient prognosis in several studies [17, 30, 31]. In our previous TMA studies on Upk1a and Upk1b we were not able to investigate the prognostic role of these proteins due to a too small cohort of patients with follow-up data [9, 10]. The extension of our cohort to now 636 patients with clinical follow-up data undergoing cystectomy for muscle-invasive urothelial cancer did not suggest a prognostic role of Upk1a/1b expression in analyses involving the entire cohort but lead to data that suggest a complex relationship between uroplakin expression and tumor aggressiveness. Complexity is supported by a significant association of Upk1a and Upk1b positivity with both nodal metastasis and tumor infiltration of lymph vessels and the stage-dependent relationship with patient outcome showing a statistically significant association of combined Upk1a/Upk1b expression with poor prognosis in pT4 but not in pT2 and pT3 cancers. That the combination of Upk1a/Upk1b and GATA3 resulted in an even stronger association with patient survival and that this link was still completely limited to pT4 cancers may suggest that IHC panels identifying luminal type pT4 carcinomas may have clinical utility in the future.

We had earlier observed a similar stage specific link to patient outcome for CK20 expression, another molecular feature of luminal differentiation of urothelial carcinomas [32]. While it cannot be excluded that an unspecified selection bias exists for our group of pT4 carcinomas, a stage dependent prognostic role of molecular features could potentially be explained by a variable, stage-dependent efficiency of different treatment modalities on different molecular subgroups of urothelial carcinomas. For example, it could be speculated, that systemic chemotherapy is more effective in “aggressive” non-luminal urothelial carcinomas resulting in a better prognosis of these tumors in stage pT2 and pT3 while these treatments might—on average—be less effective in pT4 carcinomas due to a higher tumor burden [33]. In such a scenario, when the treatment lacks efficiency, the more favorable “luminal” carcinomas may have a better prognosis then the more aggressive “non-luminal” cancers. An analogous scenario has been reported for breast cancer. In this tumor, HER2 amplification was earlier found to be strongly linked to poor patient prognosis [34] while it is now rather a predictor of good prognosis due to the striking effect of anti-HER2 therapies [35]. Studies are now needed to investigate the predictive rather than the prognostic role of molecular subtypes of urothelial carcinomas.

In summary, the results of our study demonstrate that Upk1a and/or Upk1b IHC can well complement GATA3 for the distinction of urothelial carcinomas. They also reveal a progressive loss of Upk1a/Upk1b expression during stage progression and a strong prognostic role of GATA3/Upk1a/Upk1b expression limited to pT4 carcinomas.

### Supplementary Information

Below is the link to the electronic supplementary material.

Supplementary file1 Figure 1 Combined Upk1a and Upk1b immunostaining and patient prognosis (PDF 99 KB)

Supplementary file2 Figure 2 Upk1a, Upk1b, and GATA3 in urothelial carcinomas. The panels show Upk1a/b and GATA3 immunostaining results in two pT2-4 urothelial carcinomas. In one tumor, a significant Upk1a (A) and Upk1b (B) staining is seen while GATA3 is negative (C). The other pT2-4 carcinoma lacks Upk1a (D) and Upk1b (E) staining but shows a strong GATA3 positivity (F). The images A–C and D–F are from consecutive tissue sections (PDF 487 KB)

Supplementary file3 Table 1 Patient cohort (DOCX 13 KB)

## Data Availability

All data generated or analyzed during this study are included in this published article [and its supplementary information files].
