# Supplementary figures and images for: Loss of Upk1a and Upk1b expression is linked to stage progression in urothelial carcinoma of the bladder
Source: Int Urol Nephrol. 2023 Oct 1;56(2):499–508. doi: 10.1007/s11255-023-03800-0 (PMC10808463; doi:10.1007/s11255-023-03800-0)

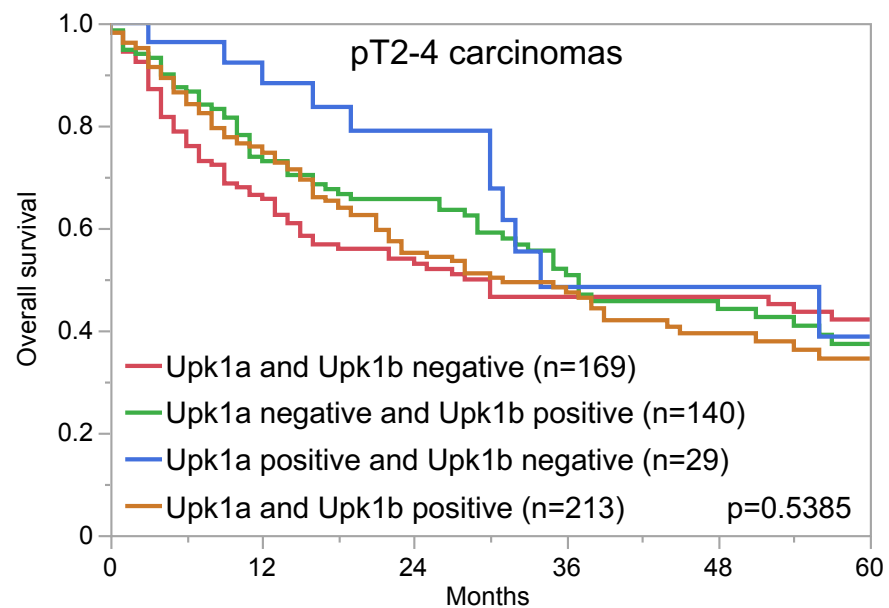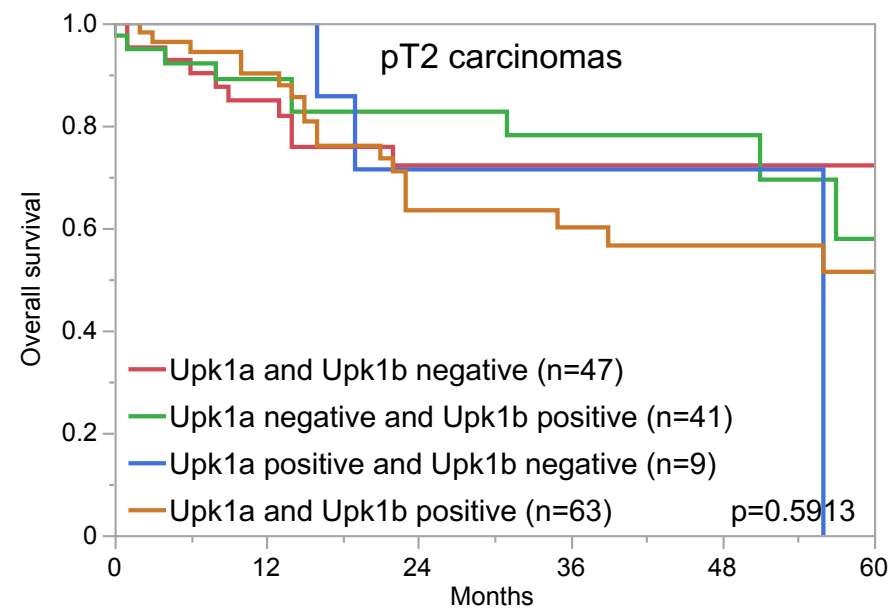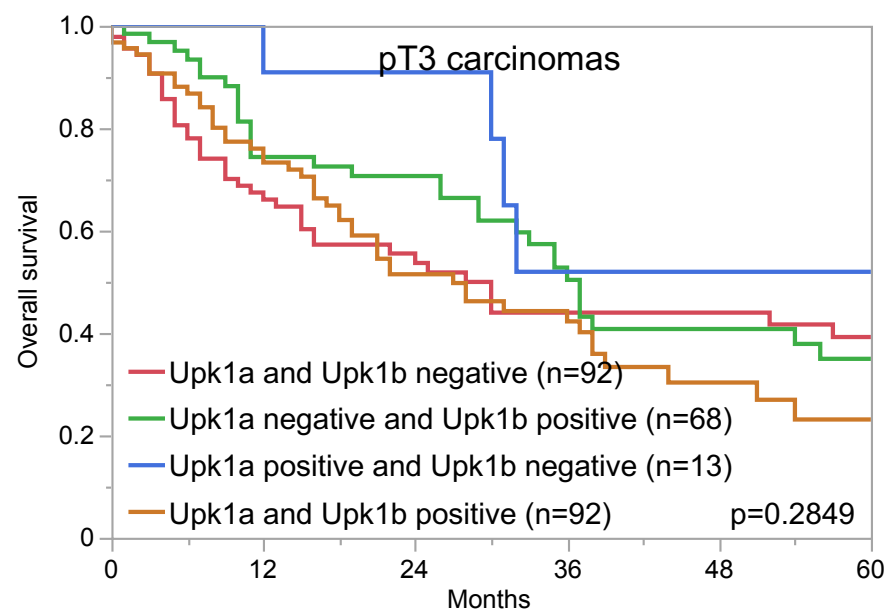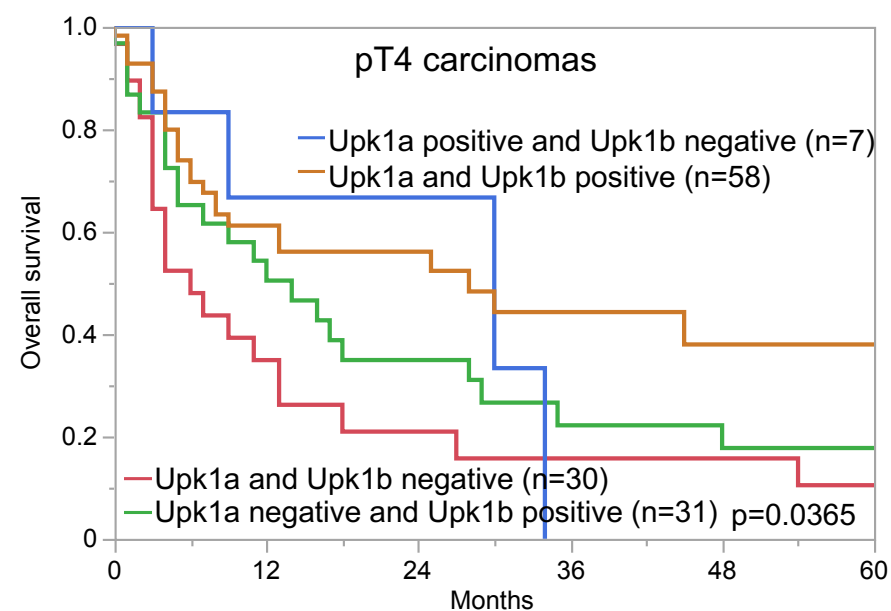

Supplement: Supplementary file 1 — Supplementary file1 Figure 1 Combined Upk1a and Upk1b immunostaining and patient prognosis (PDF 99 KB) [file 11255_2023_3800_MOESM1_ESM.pdf]

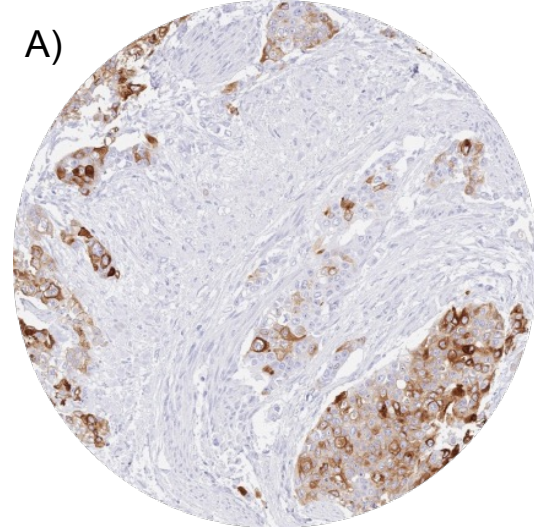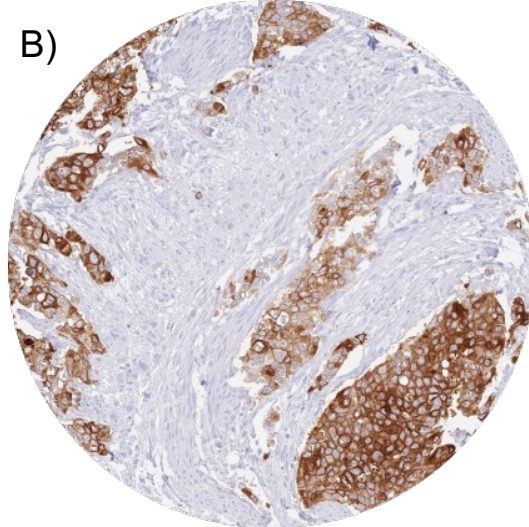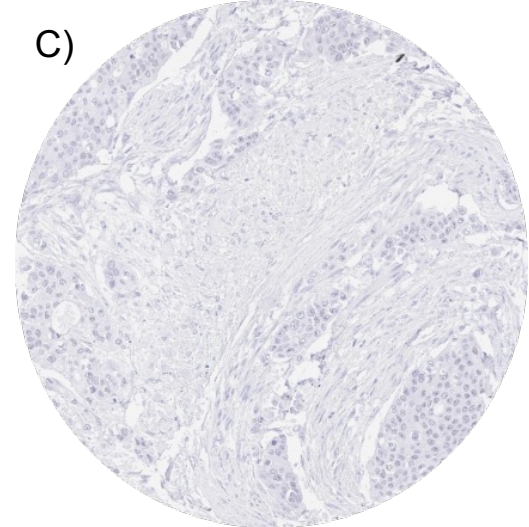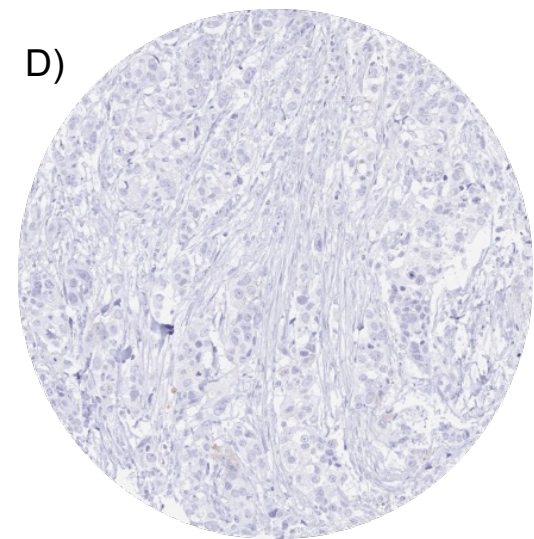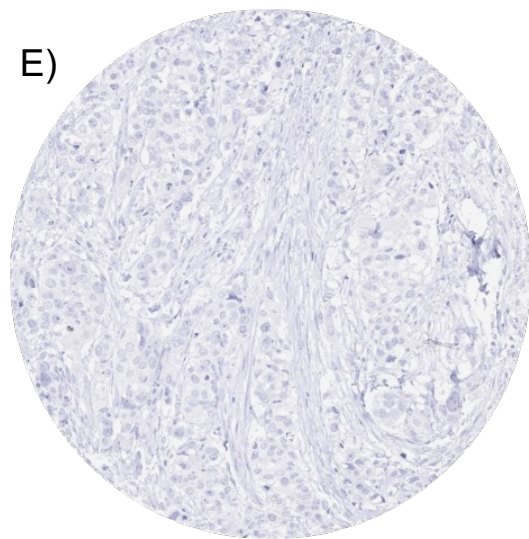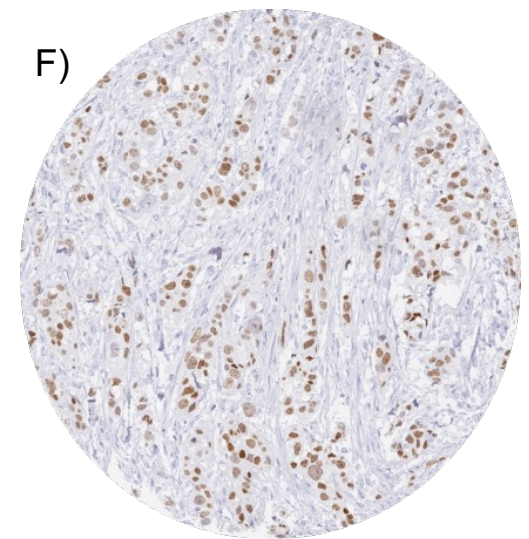

Supplement: Supplementary file 2 — Supplementary file2 Figure 2 Upk1a, Upk1b, and GATA3 in urothelial carcinomas. The panels show Upk1a/b and GATA3 immunostaining results in two pT2-4 urothelial carcinomas. In one tumor, a significant Upk1a (A) and Upk1b (B) staining is seen while GATA3 is negative (C). The other pT2-4 carcinoma lacks Upk1a (D) and Upk1b (E) staining but shows a strong GATA3 positivity (F). The images A–C and D–F are from consecutive tissue sections (PDF 487 KB) [file 11255_2023_3800_MOESM2_ESM.pdf]
