# Supplementary material for: Loss of Upk1a and Upk1b expression is linked to stage progression in urothelial carcinoma of the bladder
Source: Int Urol Nephrol. 2023 Oct 1;56(2):499–508. doi: 10.1007/s11255-023-03800-0 (PMC10808463; doi:10.1007/s11255-023-03800-0)
Supplement: Supplementary file 3 — Supplementary file3 Table 1 Patient cohort (DOCX 13 KB) [file 11255_2023_3800_MOESM3_ESM.docx]

|  | **study cohort on TMA (n=2710)** |
| --- | --- |
| **follow up** | 636 |
| months |  |
| mean | 26.7 |
| median | 15.0 |
| **pathological tumor stage** | |
| pTa | 887 (38.4%) |
| pT1 | 49 (2.1%) |
| pT2 | 462 (20.0%) |
| pT3 | 615 (26.6%) |
| pT4 | 298 (12.9%) |
| **tumor grade** | |
| G2 | 820 (30.6%) |
| G3 | 1858 (69.4%) |
| **pathological lymph node status** | |
| pN0 | 734 (62.0%) |
| pN+ | 449 (38.0%) |
| **resection margin status** | |
| R0 | 595 (80.6%) |
| R1 | 143 (19.4%) |
| **lymphatic vessel infiltration** | |
| L0 | 275 (49.5%) |
| L1 | 281 (50.5%) |
| **blood vessel infiltration** | |
| V0 | 450 (74.4%) |
| V1 | 155 (25.6%) |
| Percent in the column "study cohort on TMA" refers to the fraction of samples across each category. Numbers do not always add uo to 2,710 in the different categories because of cases with missing data. | |
